# Plasmonic wearable adhesive patch for a SERS-based sweat sensor†

**DOI:** 10.1039/d5ra00529a

**Published:** 2025-04-17

**Authors:** Vineeth Puravankara, Anusree Morayi, Swithin Hanosh, Sajan D. George

**Affiliations:** a Manipal Institute of Applied Physics, Manipal Academy of Higher Education Manipal 576104 India sajan.george@manipal.edu; b Centre for Applied Nanosciences, Manipal Academy of Higher Education Manipal 576104 India

## Abstract

The development of flexible and wearable patches made from biocompatible materials for the molecular fingerprinting of body fluids is an emerging area of research in the field of healthcare devices. Herein, a surface-enhanced Raman scattering sensor was designed and developed using a two-layer paper-based substrate: the first layer was mixed with polydimethylsiloxane and oleic acid for skin adhesion, and the second layer comprised a filter paper with *in situ* reduced nanoparticles for Raman signal enhancement of sweat components. The design of the patch avoids the direct exposure of nanoparticles and the excitation laser to the skin. The volume ratio of polydimethylsiloxane to oleic acid in the adhesive mixture was optimized for maximum adhesion to various substrates with oil residue. The plasmonic paper employed here exhibited excellent limits of detection of 55.9 μM and 47.8 μM for the sweat components urea and lactate, respectively. By utilizing the skin-adhesive patch, the multiplexed detection of urea and lactate could be achieved directly from sweat using surface-enhanced Raman spectroscopy. The developed SERS patch can be potentially utilized as a wearable healthcare sensor for the molecular fingerprinting of body fluids and opens avenues for the development of such sensors in the field of wearable personalized healthcare devices.

## Abstract 2

The development of flexible and wearable patches made from biocompatible materials for the molecular fingerprinting of body fluids is an emerging area of research in the field of healthcare devices.

## Graphical abstract

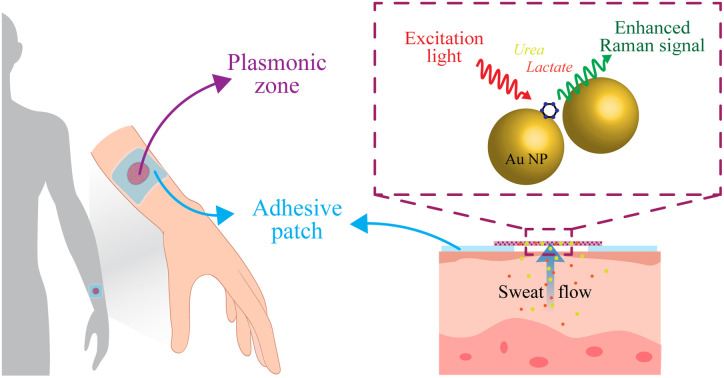

## Introduction

The increasing requirement for real-time health monitoring, particularly for probing non-communicable diseases, *via* flexible and wearable sensors has led to intense research in the utilization of body fluids for such purposes.^1–3^ Although blood testing is still considered the gold standard for obtaining reliable health data, periodical blood monitoring often remains a challenge owing to the inconvenience and difficulty in obtaining blood samples from subjects such as neonates and aged people. Researchers are now focusing on the development of the so-called “3^rd^ wave” of wearable biosensing. In the 3^rd^ wave of sensing, the focus is on the development of platforms for the continuous monitoring of analytes from body fluids, such as sweat.^4^ By their integration with established sensing modalities, wearable sensors have been shown to have the potential to measure several human body parameters, including the heart rate, blood oxygenation, body temperature, motion, stiffness, and strain.^5–12^ Knowing the level of sweat components, such as urea and lactate, is important as it can provide valuable insights into human health and fitness. For example, the level of urea is considered a biomarker as a variation in the level can indicate kidney dysfunction and the hydration status. A level of urea around 50 mM in sweat is reported to reflect kidney dysfunction, such as uremia, and previous studies have illustrated that dehydration can lead to a variation in the urea level to around 27.8 mM.^13,14^ Alternatively, the level of lactate in sweat gives information about muscle fatigue and overall energy metabolism during physical activity. Therefore, by tracking lactate and urea levels, individuals can better understand their physiological responses to exercise and overall health status. Sweat is of high clinical value as it contains metabolites, exogenous non-metabolic agents, hormones, electrolytes, and other components.^4^ Probing sweat for the diagnosis of cystic fibrosis *via* monitoring sweat chloride has demonstrated great potential.^15,16^ Additionally, sweat has been used to quantify certain drugs in drug monitoring,^17^ therapeutic dosing,^18^ nutrient and vitamin quantification,^19,20^*etc.* The probing of sweat using electrochemical or non-electrochemical wearable sensors has been employed for health monitoring by probing metabolites (*e.g.* glucose and lactate), electrolytes (*e.g.* sodium, potassium, calcium, and chloride), heavy metals, ingested substances, and drugs (*e.g.* caffeine and l-dopa) in sweat.^21–26^ The development of these types of wearable flexible sweat sensors is highly significant for non-invasive, real-time health monitoring. Unlike traditional blood-based diagnostics, these sensors allow the continuous tracking of biomarkers in sweat, such as urea, lactate, glucose, and electrolytes, directly from sweat, providing valuable insights into hydration levels, personal health, and disease conditions. Their flexibility and skin-conformal design enhance user comfort, making them ideal for long-term wearing and user-friendly. Contrary to rigid sensors, flexible and wearable sensors are soft and can attach to different bio-interfaces with random shapes. Of late, massive interest has been drawn to developing a multiplexed analytical platform to probe various components of sweat as this could provide better accuracy for diagnosis.

Despite the great promise of sweat sensors, the major challenge in the field of sweat sensing is the rapid evaporation of secreted small amounts of sweat. Therefore, it is of paramount importance to develop sensing platforms that can detect the biomarkers of interest from an ultra-low sample volume. The major sensing platforms that are currently employed depend on colorimetry, chronoamperometry, cycle voltammetry, differential pulse anodic stripping voltammetry, differential pulse voltammetry, electrochemical impedance spectrometry, open circuit potential, or square wave anodic stripping voltammetry.^27–31^ Though electrochemical sensors are a popular choice for wearable sensing technologies owing to their rapid and sensitive detection with good compatibility, their complex fabrication and low selectivity for the simultaneous detection of multiple biomarkers are some of the major challenges. Recent advances in nanotechnology and photonics have facilitated the development of the surface-enhanced Raman scattering (SERS)-assisted spectroscopic probing of sweat components using portable Raman spectrometers in a label-free manner.^32–34^ SERS utilizes the huge enhancement in the electromagnetic field at hot spots due to the plasmonic enhancement of the substrate (commonly metallic) to amplify the fingerprint signature of an analyte.^35–39^ The huge enhancement in the electromagnetic field (∼10^11^-fold) facilitates the detection of an ultra-low concentration of the target analyte, while the fingerprinting capability of the technique enables selective and multiplexed detection.^39^ The integration of microfluidic channels, including paper microfluidic technologies and optical fibers, with SERS techniques has initiated extensive research in the area of SERS for probing body fluids, including sweat.^17,40^ Using the SERS technique, various biomarkers in sweat, such as lactic acid, urea, creatine, and nicotine, have been investigated in detail.^40–42^ However, most of the wearable SERS-based platforms involve complex fabrication protocols that make them less cost-effective and affect the reproducibility of the substrate.^43,44^ Reproducibility is crucial for ensuring accurate and reliable detection across multiple uses, which is especially challenging in wearable applications where flexible substrates and exposure to varied conditions can further impact the performance.^45–47^ Addressing these reproducibility challenges requires optimized and standardized fabrication techniques to maintain a consistent SERS enhancement and signal uniformity in wearable sweat sensors. In this context, paper-based SERS substrates are emerging as promising candidate for sweat component detection and measurement. The adoption of a paper-based substrate offers the advantage of high wettability (liquid absorption) and transport capability (wicking) that facilitate the instantaneous uptake of sweat upon secretion to the probing zone. This reduces the system response time and limits sweat evaporation loss.

Herein, we report a wearable SERS patch that allows the multiplexed detection of urea and lactic acid from sweat. The wearable patch included two layers of paper, in which one layer was made of skin and a paper adhesive *via* the incorporation of a biocompatible polydimethylsiloxane (PDMS)–oleic acid (OA) mixture, while the second layer was made plasmonic *via* an *in situ* reduction of plasmonic gold nanoparticles onto the paper substrate. The PDMS–OA ratio was tailored to obtain maximum adhesive strength against various substrates, including metallic, plastic, concrete, and skin. A hole was made in this adhesive patch in the side to be attached to the skin, while the other side was attached to the plasmonic paper to avoid the direct contact of the plasmonic particles with the skin. After independently measuring the limit of detection for the sweat components urea and lactic acid the patch was employed for the sensing of these components directly from the sweat, demonstrating sensitivity in detecting the sweat components. The developed adhesive paper-based SERS patch thus holds promise for widespread applications in wearable SERS sensors, facilitating the real-time monitoring of drug levels and illegal substances as well as enable comprehensive body fluid analysis. Moreover, the facile and biocompatible design of the adhesive patch enables its potential adaptation to a wide range of physiological and environmental sensing applications, making it a valuable tool in personalized healthcare.

## Experimental

### Chemicals and materials

Poly(dimethylsiloxane) (PDMS) (Sylgard 184 silicone) was purchased from Dow Corning. Oleic acid (OA), gold chloride (III) trihydrate ≥99% (HAuCl_4_·3H_2_O), rhodamine 6G (95%), urea, sodium hydroxide (NaOH) and d-glucose were purchased from Sigma-Aldrich Co., Ltd. Calcium lactate was purchased from Molychem. Standard A4-sized paper was purchased from local supermarkets.

### Fabrication of the adhesive patch

**Fig. 1:**
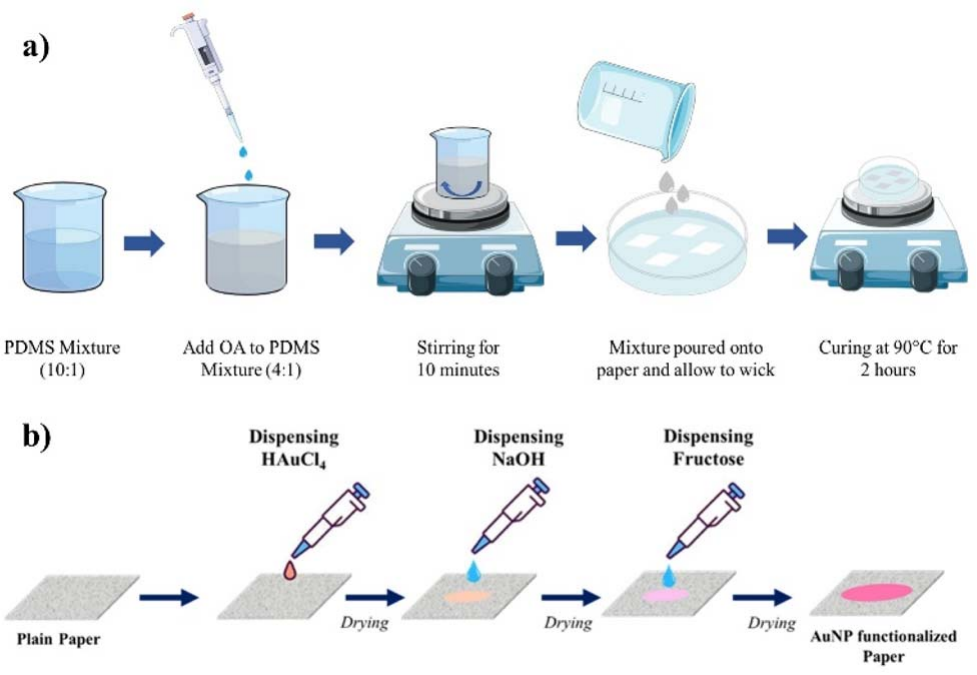
(a) Schematic of the fabrication of the PDMS–OA adhesive patch. (b) Schematic of the fabrication procedure of the *in situ* reduction of AuNPs onto the paper substrate.

A biocompatible adhesive paper-based wearable patch was developed using the PDMS and OA. For this, initially, a mixture of PDMS was prepared by mixing a Sylgard 184 elastomer base and curing agent in a ratio of 10 : 1. The mixture was thoroughly mixed with a stirrer and was desiccated for 30 min to remove the air bubbles formed during the mixing. Subsequently, OA was added to this PDMS mixture in a ratio of 4 : 1, with 4 parts PDMS and 1 part OA, then thoroughly mixed to achieve homogeneity. This addition of OA caused a noticeable change in the mixture's appearance, shifting from a transparent state to a turbid and cloudy state. This turbid mixture was then further diluted with 50% *n*-hexane. Paper pieces with the dimension 5 × 5 cm were cut from a standard A4 sheet and placed in a clean Petri dish. Subsequently, the diluted PDMS–OA mixture was carefully poured onto the paper pieces in the Petri dish. The coated paper substrates were held upright to allow the excess mixture to drip off and were then cured at 90 °C for 2 h. Fig. 1a shows a schematic of the fabrication process. After curing, the adhesion performance of the resulting adhesive patch was evaluated.

### Optimization of the PDMS:oleic acid ratio

In preparing the PDMS–OA mixture for the fabrication of the adhesive patches, the ratio of PDMS to oleic acid is a significant factor, and it determines the strength of adhesiveness. Therefore, optimizing and determining the optimized ratio of PDMS and OA is important for achieving optimal adhesion while preventing an excess oil content on both the paper substrate and the skin. For this, we evaluated different ratios of the PDMS and OA such as 4 : 0.5, 4 : 1, 4 : 2, 4 : 3, and 4 : 4. The adhesion properties of these patches were evaluated and compared for assessing the optimum adhesion properties.

### Characterization of the developed PDMS/OA adhesive patch

The developed adhesive patch was characterized using Fourier-transform infrared (FTIR) spectrometry (JASCO FTIR-6300) to identify the presence of functional groups on its surface. The adhesion performance of the prepared PDMS/OA patch was evaluated using different types of materials, such as concrete walls, wood, plastic, metal, and skin. Furthermore, to examine the load-bearing capacity of the fabricated adhesive patch, we tested its ability to hold various loads ranging from 1 gram to 50 grams, thereby determining its load-bearing capacity and the maximum weight it can support without detachment. To quantify the value of adhesion force, tear strength measurements were carried out using a universal testing machine (Instron 3366). Water contact angle (WCA) measurements were carried out using a commercial contact angle instrument (Holmarc).

### Fabrication of plasmonic paper-based substrates for the SERS studies and modification of the adhesive patch

For carrying out the SERS studies of sweat components, a plasmonic paper substrate was fabricated *via* an *in situ* reduction of gold nanoparticles (AuNPs) onto the paper. Initially, circular paper pieces with a diameter of approximately 1 cm were cut out. A 10 μL droplet of 30 mM HAuCl_4_·3H_2_O solution was then dispensed onto the paper. Subsequently, 10 μL of NaOH solution was added and allowed to air dry. To facilitate the *in situ* reduction of AuNPs onto the cellulose fibers, a 10 μL droplet of glucose solution was added. The paper changed color from white to wine red, indicating the formation of Au nanoparticles. Fig. 1b shows a schematic of the fabrication procedure involving the *in situ* reduction of AuNPs onto the paper substrate.

The developed plasmonic paper substrates were further incorporated into a PDMS/OA wearable adhesive patch. Holes with a 5 mm diameter were made on the PDMS/OA patch. Circular plasmonic paper substrates, 8 mm in diameter, were then cut and integrated into the adhesive patches by placing them over the holes. In our experiments, only one hole was made per substrate. ESI Fig. S1† shows the developed Au-PDMS/OA adhesive patch. This modified plasmonic wearable adhesive patch, hereafter referred to as Au-PDMS/OA, was used for sweat component analysis using the SERS technique.

### Characterization of the AuNP attached paper substrate

To confirm the AuNPs had been reduced onto the paper, diffuse reflectance spectra were recorded using a UV-visible-NIR spectrophotometer (Lambda 750, PerkinElmer) for the plain paper and the AuNP-reduced paper. Field emission scanning electron microscopy (FESEM) was carried out to investigate the surface morphology and confirm the presence of plasmonic nanoparticles on the paper surface.

### Raman spectroscopic measurements using the AuNP-attached paper substrate

Raman spectroscopic studies of the sweat components using the developed Au-PDMS/OA patch were carried out using a commercial portable fiber-based Raman setup (iRaman plus, B&W Tek). This system has a fiber-based Raman setup with an excitation wavelength of 785 nm and a CCD detector with high quantum efficiency. The laser was coupled through an optical fiber probe, which acts as an excitation and Raman signal collection probe. Here, the sample was kept in a manual *X*–*Y*–*Z* translation stage. A laser line filter was employed here to eliminate the side bands and guarantee a narrow excitation wavelength for the sample by eliminating all the secondary excitation lines. Subsequently, the Raman scattered signals from the sample underwent filtration using a notch filter and were recorded using a spectrograph-CCD detector. For SERS studies, a 10 μL droplet of the urea solution and calcium lactate solution was dispensed onto the plasmonic region of the Au-PDMS/OA patch. Raman spectral measurements were carried out in five different hydrophilic regions to confirm the reproducibility of the measurements and to calculate relative standard deviation (RSD) values. The SERS signals from the sweat components urea and lactate were collected with a laser power of 30 mW and an exposure time of 40 seconds.

## Results and discussion

### Characterization of the developed PDMS/OA adhesive patch

Initially, the adhesive nature of the fabricated PDMS/OA paper was tested against various substrates, including concrete, paraffin film, wood, plastic, glass, steel, and paper. It was observed that the adhesive paper could stick to all the substrates investigated here. It is pertinent to note here too that the incorporation of the clear two-component PDMS mixture into the cellulose paper matrix *via* dipping, and subsequent polymerization *via* thermal curing at 90 °C for 2 h did not result in an adhesive nature towards skin (ESI Fig. S2†). In our study, the PDMS–OA curing process was found to be optimum when performed at 90 °C for 2 h. Curing at a lower temperature or for a shorter duration resulted in an incompletely cured patch with a sticky texture and residual oil, which could affect adhesion. In addition, PDMS incorporation onto the paper resulted in a hydrophobic flexible paper with a water contact angle ∼105° for a 5 μL droplet. In the case of adhesive paper, the PDMS/OA ratio was varied at 4 : 0.5, 4 : 1, 4 : 2, 4 : 3, and 4 : 4 to determine the optimum OA component ratio in the mixture. It was observed when the ratio was less than 4 : 1, the substrate was less adhesive in nature, while the substrate with a ratio equal to or greater than 4 : 2 always left an oil residue on the substrate. The residual wetting of oil onto the paper substrate following the contact with the adhesive patch with different PDMS/OA ratios is shown in ESI Fig. S3.† Therefore, for the further studies, the sample prepared in the ratio 4 : 1 was employed. As observed, the direct mixing of the PDMS and oleic acid in different ratios also resulted in a sticky product, but the polymerization onto the paper matrix provided a flexible and scalable substrate for future applications.

**Fig. 2:**
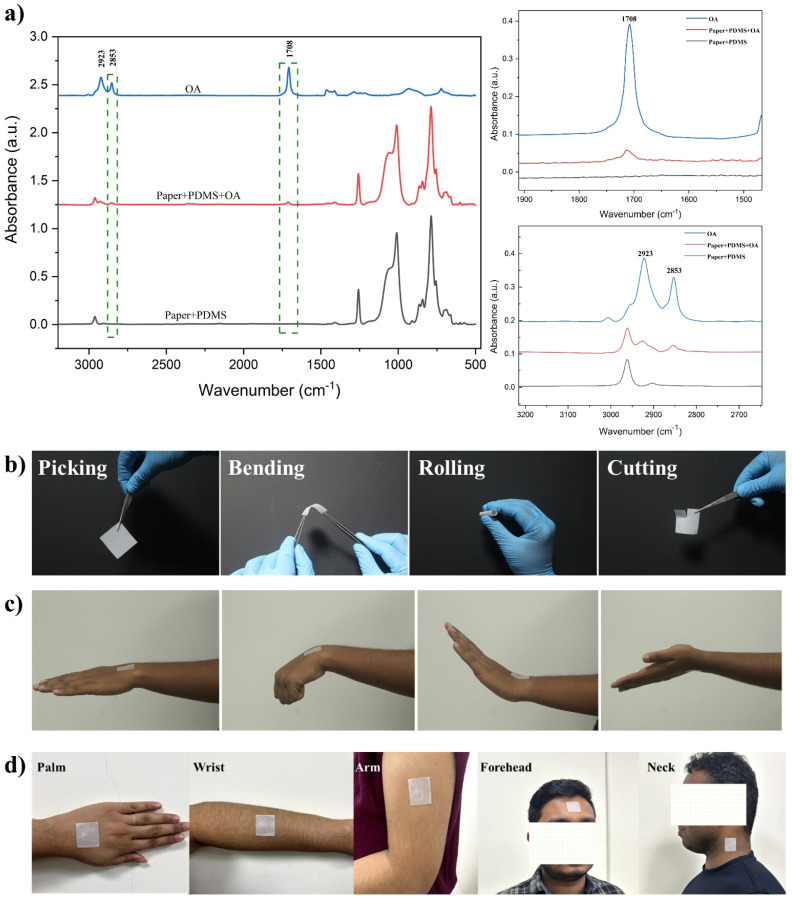
(a) ATR-FTIR spectra of the developed adhesive patch and its expanded view. (b) Photographs showing its excellent adhesion properties towards different materials. (c) Photographs showing the excellent mechanical flexibility of the prepared wearable adhesive patch. (d) Photograph of the wearable patch showing strong adherence to human skin, even during dynamic wrist movements.

The adhesive nature of the substrate originated from the partial polymerization of PDMS due to the presence of OA during the polymerization process. It has been reported that by manipulating the cross-linking network of PDMS by modifying the amount of the curing agent, adjusting thermal curing conditions, or adding chemicals, the adhesive nature of PDMS can be controlled.^48,49^ It has also been reported that, as in the present case, the hydrosilylation of PDMS can be interrupted by inhibitors when the prepolymer is mixed with oligomers, surfactants, or amine-based materials.^50,51^ The presence of oleic acid during the polymerization process resulted in a higher proportion of un- or low-cross-linked polymers in the matrix compared with highly cross-linked polymers, which resulted in a higher adhesive nature. This was further corroborated by surface wettability studies on the paper substrate cured with PDMS/OA in the ratio of 4 : 1 and the PDMS substrate. While the PDMS-cured surface exhibited a water contact angle (WCA) of 105° ± 2° for a 5 μL droplet compared with complete wetting in the case of the plain paper (WCA < 5°), PDMS/OA in the ratio of 4 : 1 showed a value of 68° ± 2°, as shown in ESI Fig. S4.† In addition, the OA in the developed substrate was further confirmed *via* ATR-FTIR spectral studies (Fig. 2a). The FTIR spectrum of the pure OA showed prominent bands located at wavenumbers of 2923, 2853, and 1708 cm^−1^; these peaks corresponded to asymmetric –CH_2_ stretching, symmetric –CH_2_ stretching, and asymmetric –C=O stretching, respectively.^52^ As observed in Fig. 2a, compared with the PDMS-incorporated paper substrate, the substrate of interest showed additional distinct peaks of OA situated at wavenumbers of 2923, 2853, and 1708 cm^−1^. This confirmed the presence of OA in the developed adhesive patch.

The adhesive nature of the chosen PDMS/OA (4 : 1) paper against various materials, such as glass, concrete wall, wood, plastic, and metal, was tested, and it was observed that the substrate adhered strongly to all the substrates (ESI Fig. S5†). The adhesive force with our substrate with normal A4 paper was probed here as an example by testing the ability of the adhesive patch with a dimension of 3 × 3 cm to hold various loads, ranging from 1 to 50 g. ESI Fig. S6† shows the load-bearing capacity of the adhesive patch. It was observed that the patch could support a weight up to 20 g, beyond which the patch started to show the detachment signature. A rough estimation of the force was made using the equation *F* = *mg*, where *m* is the mass and *g* is the acceleration due to gravity (approximately 9.8 m s^−2^), whereby the adhesion force was calculated to be approximately 1.8 N. In addition, tear strength measurements on various substrates were carried out using a commercially available Instron 3366 model universal testing machine. The instrument features two loading levers: one at the top and one at the bottom, whereby one end of the adhesive patch was attached to the top clamp while one end of the substrates was secured to the bottom clamp. The tear strength measurements were performed on various materials, including plastic, Teflon, paper, PDMS (polydimethylsiloxane), and metal. The tear strength measurement results revealed the maximum force of adhesion for each material was follows: plastic: 0.19 N, Teflon: 0.31 N, PDMS: 0.30 N, paper: 0.26 N, metal: 0.37 N. These values indicate the varied adhesion strengths of the patch across different substrates, with the metal substrate demonstrating the highest maximum force required for detachment, followed by Teflon, PDMS, paper, and plastic. The mechanical deformation influences on the developed wearable PDMS/OA adhesive patch were further evaluated. As demonstrated in Fig. 2b, the adhesive patch material still maintained firm and conformal healing without any tearing or shedding regardless of bending, rolling, *etc.*, showing the excellent mechanical flexibility of the prepared wearable adhesive patch. Moreover, the patch could be easily cut using scissors or a knife, allowing am ease of fabrication in any desired size and shape. In addition, the developed patch demonstrated strong adherence to human skin, maintaining close conformal contact, even during dynamic wrist movements, as illustrated in Fig. 2c. In addition, the developed patch also demonstrated strong and consistent adhesion across various body sites, including the palm, wrist, arm, forehead, and neck, as shown in Fig. 2d. We also tested the adhesive nature of the fabricated adhesive patch after 30 min and 1 h of intense exercise. The patch demonstrated strong adhesion even after rigorous physical activity, with the corresponding images showcasing its adhesive performance provided as ESI Fig. S7.† More importantly, no instances of oil residue, irritation, or allergic reactions were observed upon removal of the patch from the skin.

**Fig. 3:**
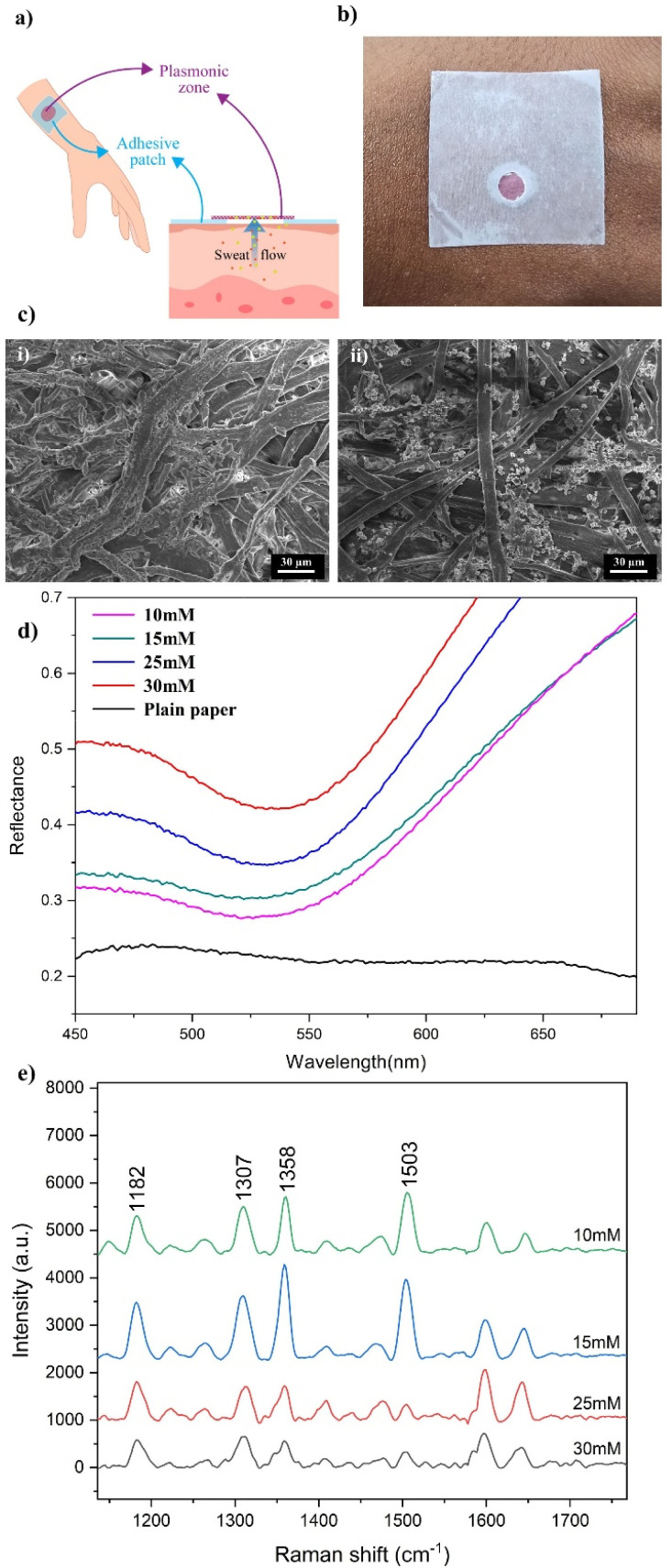
(a) Schematic representation of the Au-PDMS/OA plasmonic wearable adhesive patch. (b) Photograph of p Au-PDMS/OA. (c) FESEM images of the (i) plain paper and (ii) AuNP *in situ*-reduced paper substrate. (d) Diffuse reflectance spectra of AuNP-reduced paper substrates with different concentrations of HAuCl_4_. (e) Comparison of the Raman spectra of Rh6G recorded on substrates with different concentrations of HAuCl_4_.

In order to develop a skin-adhesive analytical platform that is based on plasmonic nanoparticles, as mentioned, a two-step process was followed here. Initially, the plasmonic filter paper was fabricated *via* the *in situ* reduction of gold nanoparticles (AuNPs) onto cellulose fibers of the paper substrate (Fig. 1b). The fabricated plasmonic paper pieces were then further integrated into the adhesive patches. Fig. 3a shows a schematic representation of the modified Au-PDMS/OA plasmonic wearable adhesive patch. Herein, a gold salt solution (HAuCl_4_) was reduced onto the cellulose fibers by glucose when the analytes were dispensed as droplets. The aldehyde functional group in glucose facilitated the reduction of Au(iii) ions in HAuCl_4_ to their Au (0) oxidation state by donating electrons.^53,54^ The synthesis of Au nanoparticles (AuNPs) began with mixing an aqueous solution of HAuCl_4_, containing AuCl_4_^−^ ions, with NaOH to adjust the pH to 10–11 to induce hydrolysis of Au(iii) chloride complex ions. The reaction can be described by the following equation:

1 [AuCl_4_]^−^ + *n*OH^*−*^ → [AuCl_4−n_(OH_*n*_)]^−^ + *n*Cl^−^

where *n* is the number of Cl^−^ ligands exchanged by [OH]^–^ ions. Upon the addition of d-glucose to the hydrolyzed precursor solution, the reduction reaction begins with the opening of the sugar ring *via* abstraction of the α-proton from the oxygen atom, leading to the oxidation of glucose to gluconic acid.^51^ This reaction causes a visible color change on the paper substrate where the reagents are dispensed, shifting from white to purple/red color, as shown in Fig. 3b. Additionally, the optical microscopic image revealed *s* significant color variation in the nanoparticle-reduced paper compared with the plain paper under similar illumination conditions, confirming the presence of nanoparticles attached to the cellulose fibers (ESI Fig. S8†). This confirmed the reduction of Au(iii) ions by glucose governed by the following chemical equation:

2 [AuCl_3_(OH)]^*−*^ + C_6_H_12_O_6_ → Au_colloid_ + Products

During the fabrication process, the paper substrate was allowed to dry at each stage of reactant addition to ensure a proper formation and adherence of the nanoparticles onto the cellulose fibers. Also in our studies, for the *in situ* nanoparticle reduction, the synthesis was performed at room temperature to ensure the controlled growth on the cellulose fibers. While higher temperatures typically accelerate the reaction rate, leading to faster nucleation and growth of nanoparticles, this could introduce variability in the nanoparticle size and distribution. The field-enhanced scanning electron microscopic image studies (Fig. 3c) clearly illustrated the presence of AuNPs on the substrate compared to the plain paper. Further, to study the effect of the reagent concentrations on the reduction mechanism, the reaction here was carried out with varying concentrations of HAuCl_4_. Four substrates were fabricated with HAuCl_4_ concentrations of 30, 25, 15, and 10 mM, respectively, while maintaining constant concentrations of NaOH and glucose. Following the observation of a visible color change, to confirm the presence of nanoparticles on the paper substrate, diffuse reflectance spectra were recorded for the AuNP-reduced paper substrates with different concentrations of HAuCl_4_ and for the control paper (Fig. 3d). The spectra of the AuNP-reduced paper substrates displayed a dip at 532 nm, which could be attributed to the absorption by the AuNPs, compared to the spectrum for the plain paper. Moreover, with increasing the concentration of HAuCl_4_, the intensity of this dip also increased, being highest for the 30 mM HAuCl_4_ substrate, suggesting a higher density of AuNPs reduced onto the paper. Further, to investigate the impact of varying the reagent concentrations and to optimize SERS performance on substrates with different concentrations of HAuCl_4_, studies were carried out by performing Raman spectroscopy for a fixed concentration (100 μM) of the standard dye molecule Rhodamine 6G (Rh6G) across these substrates (Fig. 3e) under the same experimental conditions (laser wavelength: 785 nm, laser power: 30 mW and integration time: 10 s). It was observed that the substrate with 15 mM of HAuCl_4_ demonstrated superior performance in terms of signal intensity compared to the others. Consequently, we selected the substrate with this set of concentration of reagents for the further studies. Further, to evaluate the uniformity of the Raman signal on the Au-functionalized paper region, we recorded the Raman spectra of 10 μL of a standard dye, Rhodamine 6G (1 mM), at 10 different spots on the plasmonic nanoparticle-incorporated paper. For estimating the RSD values, we analysed five prominent Raman peaks of Rh6G at 1083, 180, 1305, 1360 and 1505 cm^−1^. ESI Fig. S9† shows the uniformity of the Raman peaks in the spectra obtained from these 10 spots, demonstrating that the peak intensities were consistent, with only moderate variance. The RSD values were calculated and found to be less than 15%, confirming the uniformity and reproducibility of the Raman signal across the surface.

**Fig. 4:**
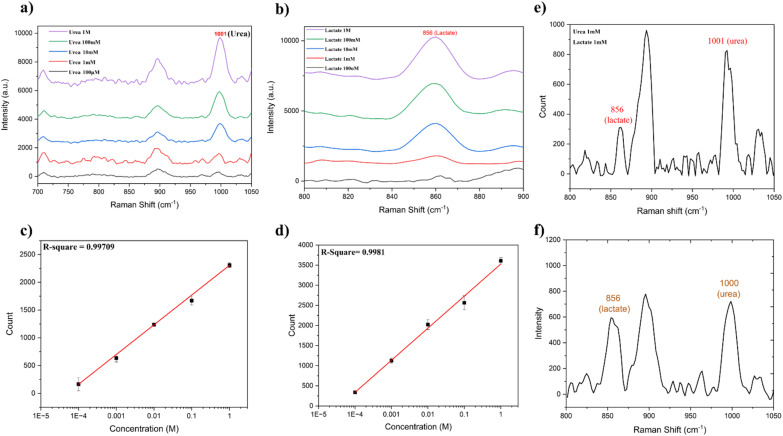
(a) Comparison of the Raman spectra of urea at different concentrations on the developed substrate. (b) Comparison of the Raman spectra of lactate at different concentrations on the developed substrate. (c) Linear variation of the 1001 cm^−1^ peak of urea *vs.* concentration. (d) Linear variation of the 856 cm^−1^ peak of lactate *vs.* concentration. (e) Multiplexed detection of urea and lactate from a mixture. (f) Raman spectra of sweat recorded using the Au-PDMS/OA patch showing the characteristic peaks of urea and lactate.

The potential of this Au-PDMS/OA adhesive patch to fingerprint the chemical identity of the sweat component molecules *via* the SERS technique was investigated by measuring the Raman spectra of urea and lactate at various concentrations. Herein, 10 μL droplets of urea and lactate solutions with different concentrations, such as 100 mM, 10 mM, 1 mM, and 100 μM, were dispensed on the developed plasmonic substrate, and Raman spectra were recorded. The recorded Raman spectra of urea and lactate at different concentrations are shown in Fig. 4a and Fig. 4b, respectively. The Raman spectra clearly illustrate the characteristic peak of urea and lactate at 1001 cm^−1^ and 859 cm^−1^, respectively. The peak at 1001 cm^−1^ corresponded to symmetric N–C–N stretching.^55,56^ The prominent peak of lactate at 856 cm^−1^ could be attributed to its characteristic ρ(CH_3_) vibrational mode.^57^ However, in our experiments, in addition to the analyte peaks, we observed some peaks that originated from the paper substrate. Therefore, for our analysis, only prominent characteristic peaks corresponding to urea and lactate were used. It is pertinent to note that the paper did not exhibit any characteristic Raman peaks at these peak positions.

As observed in Fig. 4a and b, the sweat components urea and lactate exhibited distinct Raman spectral peaks down to a concentration of 100 μM on the developed plasmonic substrate. The limit of detection (LOD) for urea was calculated by plotting the variation of intensity of the 1001 cm^−1^ Raman peak as a function of concentration (Fig. 4c). Using the expression *y* = *Y*_B_ + 3*S*_B_, where *y* is the intensity count at zero analyte concentration, *Y*_B_ is the background signal, and *S*_B_ is the standard deviation corresponding to the background, the LOD was estimated to be 55.9 μM for urea. Similarly, the LOD for lactate on the developed substrate was estimated by plotting the variation of intensity of the 856 cm^−1^ Raman peak *versus* its corresponding concentration (Fig. 4d). For lactate, the LOD was calculated to be 47.8 μM. The relative standard deviation of the measurements was found to be <15%. A comparison of the performance and detection levels of urea and lactate using various wearable SERS sensors in some of the reported literature is presented in ESI Table ST1.† This indicates that the SERS performance of the developed wearable adhesive plasmonic paper-based patch was comparable with the reported values achieved through different fabrication approaches. The estimated LOD values suggest the high sensitivity of our developed substrate toward the detection of these sweat components. Notably, these LOD values were significantly lower than the concentrations typically found in normal sweat (5–50 mM for urea^58^ and 16–30 mM for lactate^59,60^). The substrate exhibited a linear relationship between the counts and concentration within the physiologically significant ranges observed during elevated conditions, such as physical activity or certain abnormalities. The high sensitivity and Raman peak-based selectivity demonstrated the potential of our substrate for accurately detecting even low levels of these analytes, making it highly suitable for applications in non-invasive sweat analysis and the real-time monitoring of physiological conditions. Furthermore, to validate the capability of the developed plasmonic substrate for multiplexed detection, we conducted Raman spectroscopic experiments involving the simultaneous detection of both urea and lactate. For this, an equal volume (10 μL) of 1 mM concentration of urea and lactate was dispensed onto the Au-PDMS/OA substrate and the Raman spectra were measured (Fig. 4e). From Fig. 4e, it is clear that the mixture showed distinct Raman spectral features of both components: 1001 cm^−1^ for urea and 856 cm^−1^ for lactate, thus illustrating its potential for the multiplexed detection of analytes. Building on this multiplexed detection capability, we further assessed the patch's anti-interference ability to ensure precise analyte detection in complex sample environments. For this, solutions containing urea and lactate at concentrations of 1, 5, and 10 mM were prepared by mixing equal volumes of urea and lactate. Subsequently, 10 μL of each solution was dispensed onto the plasmonic paper region, and Raman spectra were recorded. As evident from ESI Fig. S10,† we can clearly see the prominent peaks of urea and lactate situated at 1000 and 856 cm^−1^, respectively. In addition to this, a substrate peak at 895 cm^−1^ corresponding to the paper was observed. However, it is important to note as the concentrations of urea and lactate increased, so did their respective peaks, whereas the substrate peak showed minimal variation. This indicates that the substrate spectra did not significantly contribute to the spectra of the sweat components, thus demonstrating the patch's anti-interference capability.

For real-life application involving the collection and monitoring of sweat components, the fabricated PDMS/OA adhesive patch was modified by incorporating the plasmonic substrate into its design, as described in the experimental section. The skin-adhesive patch, Au@PDMS/OA patch, featured a region that ensured strong adhesion to the skin, while the hydrophilic plasmonic region promoted efficient sweat transport from the skin to the plasmonic paper area. This design facilitates easy sweat collection for the analysis. For the collection of sweat, the developed Au@PDMS/OA patch was worn on the skin of a healthy volunteer, and the sweat was collected at the plasmonic region. Informed consent was obtained from the healthy human volunteers who participated in this study. Based on our observations, it took approximately 2 minutes for the sweat to fully saturate the plasmonic paper, allowing for effective detection. Therefore, to ensure consistent signal quality, spectra were recorded after a 2-minute period following patch application. SERS spectra were recorded for a laser power of 30 mW and a duration of 60 seconds using a commercial fiber-based Raman spectrometer.

To assess the liquid-collection capacity of the developed plasmonic paper with *in situ-*reduced nanoparticles, we evaluated the capillary rise and compared it with that of regular filter paper. Strips measuring approximately 2.5 cm × 1 cm were prepared from normal filter paper and plasmonic Au-reduced paper. One end of each strip was simultaneously immersed in water to observe the water rise and capillary height. It was found that both the normal filter paper and the gold-reduced filter paper exhibited similar capillary rise heights, as shown in ESI Fig. S11,† indicating that the reduction of nanoparticles does not hinder the liquid-collection capacity of the paper. The advantage here is that the plasmonic nanoparticles are not directly in touch with the skin and are separated by the adhesive paper layer. However, the secreted sweat droplet wicks the filter paper, and the laser signal is collected from the other side of the paper. This avoids direct laser exposure to the skin and thereby reduces any skin damage due to laser exposure. Fig. 4f presents the Raman spectra of sweat collected with the wearable Au@PDMS/OA patch. The spectra revealed distinct peaks at 1001 cm^−1^, corresponding to urea, and 856 cm^−1^, associated with lactate. These peaks were indicative of the successful detection of these components using our developed SERS-based adhesive patch. By referencing the linear calibration plots shown in Fig. 4c and d, the concentrations of urea and lactate in the sweat samples were estimated to be 8 mM for urea and 13 mM for lactate, demonstrating the patch's effectiveness in enabling the quantitative analysis of sweat components. In order to evaluate the stability of the developed patch, we recorded the sweat spectra using the patch at different time intervals, such as 1st, 10th, 30th, and 60th day. The results shown in ESI Fig. 12 indicate that the patch could maintain its performance, consistently displaying the prominent peaks of urea and lactate with comparable peak intensities even after 60 days. With the approval of the appropriate ethical committee, the developed patch can be explored for disease diagnosis and data collection from a large number of subjects.

## Conclusions

In this study, a wearable and flexible skin-adhesive plasmonic patch was developed and investigated for is suitability toward the measurement of sweat components using a molecular fingerprint spectroscopic technique, surface-enhanced Raman spectroscopy. The adhesive nature of the commonly employed filter paper was achieved herein by immersing the filter paper in an optimized ratio of polydimethylsiloxane and oleic acid, followed by thermal curing. Upon attaching this paper with the plasmonic filter paper obtained *via* an *in situ* reduction of plasmonic gold particles, a two-layer plasmonic adhesive patch was fabricated. The fabricated plasmonic substrate was found to exhibit limits of detection of 55.9 μM and 47.8 μM for the sweat components urea and lactate, respectively. Finally, we demonstrated the ability of the wearable adhesive patch to detect urea and lactate from human sweat samples using the SERS technique. Overall, we demonstrated a simple low-cost fabrication of a SERS adhesive patch that allows the multiplexed detection of sweat components using a portable Raman spectrometer. The developed patch, which is intrinsically simple, biocompatible, low-cost, and easy to operate, holds significant potential for commercialization in personalized health monitoring and point-of-care applications *via* the analysis of body fluids, such as tears, saliva, urine, and blood.

## Data availability

Data will be available from the corresponding author upon request.

## Author contributions

Vineeth Puravankara: writing – original draft, formal analysis, data curation, and conceptualization. Anusree Morayi: writing – original draft, writing – review & editing formal analysis, and data curation. Swithin Hanosh: writing – original draft, writing – review & editing, formal analysis, data curation, and conceptualization. Sajan D. George: writing – review & editing, writing – original draft, and supervision.

## Conflicts of interest

The authors declare no conflict of interest.

## Supplementary Material

RA-015-D5RA00529A-s001
