# Supplementary material for: Plasmonic wearable adhesive patch for a SERS-based sweat sensor
Source: RSC Adv. 2025 Apr 17;15(16):12152–61. doi: 10.1039/d5ra00529a (PMC12004224; doi:10.1039/d5ra00529a)
Supplement: RA-015-D5RA00529A-s001 [file RA-015-D5RA00529A-s001.pdf]

Supplementary Information (SI) for RSC Advances.
This journal is © The Royal Society of Chemistry 2025

Supporting Information

A plasmonic wearable adhesive patch for SERS-based sweat sensor

Vineeth Puravankara[1], Anusree Morayi[1], Swithin Hanosh[1], Sajan Daniel George*[1]

[1]Centre for Applied Nanosciences (CAN), Manipal Institute of Applied Physics, Manipal Academy of Higher Education, Manipal, India – 576104

Corresponding author email: *sajan.george@manipal.edu

Table of contents

S.No.	**Topic**	**Page No.**
S1	The Photograph of the developed Au@PDMS/OA wearable adhesive patch	2
S2	Adhesion of the Paper-PDMS and the Paper-PDMS-OA substrate onto the skin	2
S3	The residual wetting onto the paper substrate following the contact with adhesive patch with different PDMS/OA ratios	2
S4	Water contact angle images of the substrates	3
S5	Photograph of the developed PDMS/OA patch showing adhesion to different materials	3
S6	Load-bearing capacity of the adhesive patch tested with loads with different weights	3
S7	Patch showing good adhesion even after intense exercise	4
S8	Optical microscopic image of the substrates	4
S9	Evaluation of the Raman signal uniformity of Rh6G at different spots on the Au-reduced paper region	5
S10	Raman spectra of mixture urea and lactate of different concentrations	5
S11	Comparison of the capillary height using normal filter paper and Au reduced plasmonic paper substrate.	6
S12	The Raman spectra of sweat recorded with the developed Au@PDMS/OA adhesive patch for different time	6
ST1	Comparison of the analytical performance of the present work with similar reported SERS based sweat sensors.	7

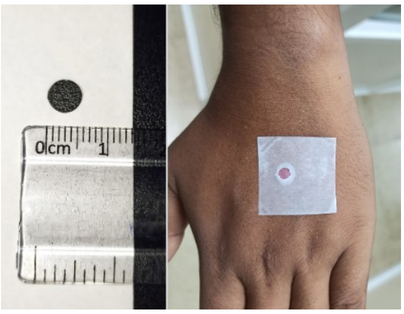

Figure S1 The Photoraph of the developed Au@PDMS/OA wearable adhesive patch

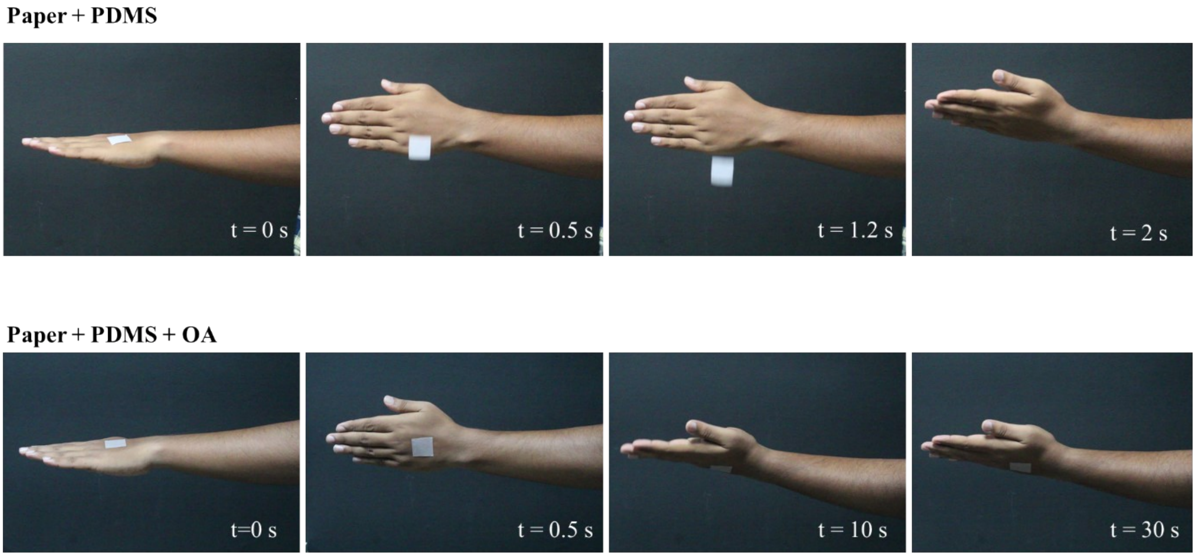

Figure S2 difference in adhesion of the Paper-PDMS and the Paper-PDMS-OA substrate on to the skin.

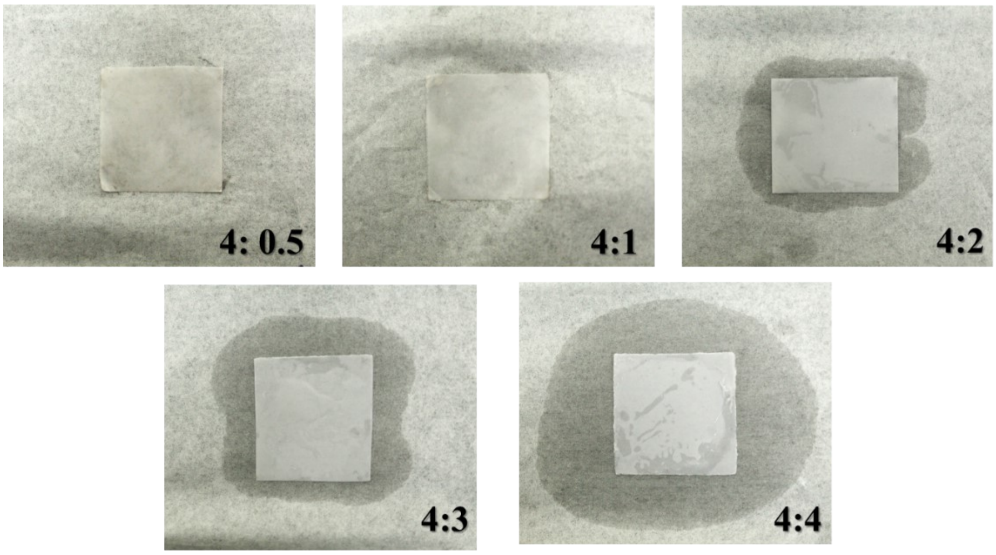

Figure S3 The residual wetting of oil onto the paper substrate following the contact with adhesive patch with different PDMS/OA ratios

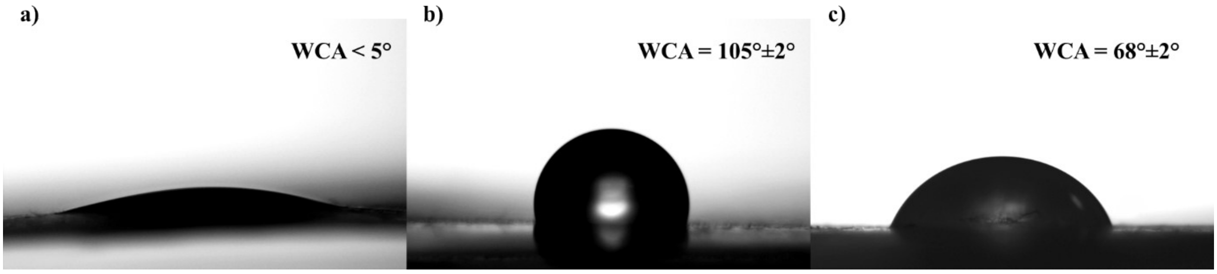

Figure S4 Water contact angle of a) plain paper b) paper-PDMS c) paper-PDMS-OA substrates

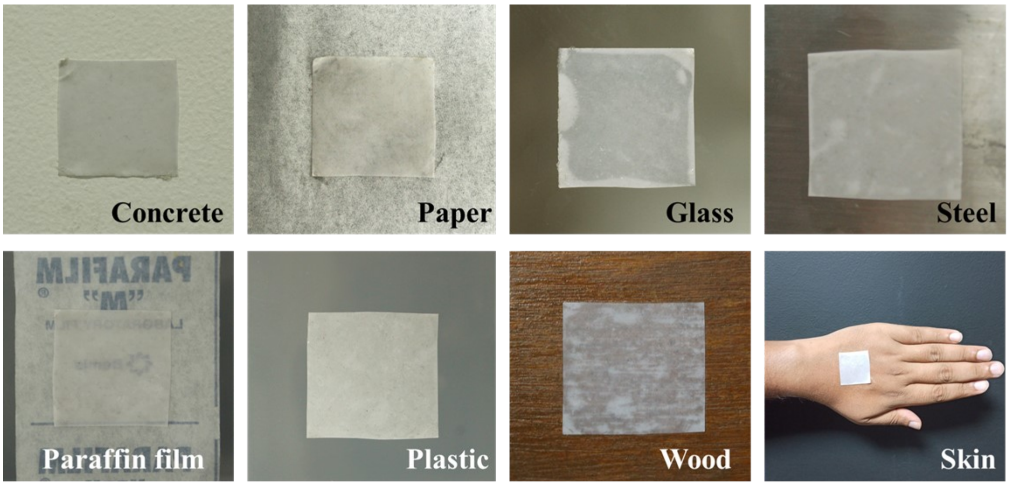

Figure S5 Photograph of the developed PDMS/OA patch showing adhesion to different materials

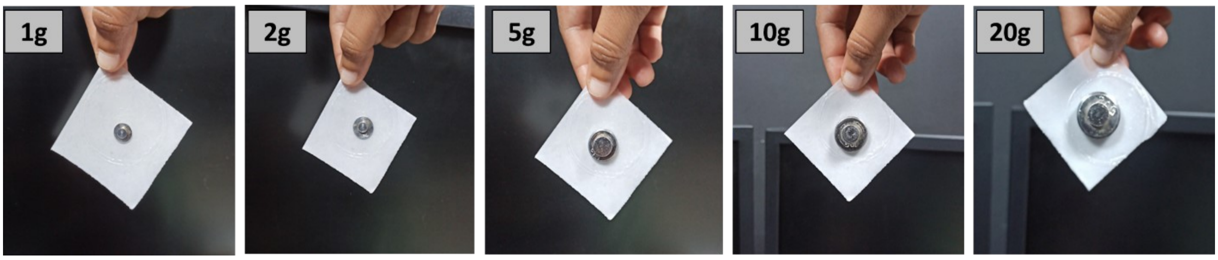

Figure S6 load-bearing capacity of the adhesive patch tested with loads with different weights

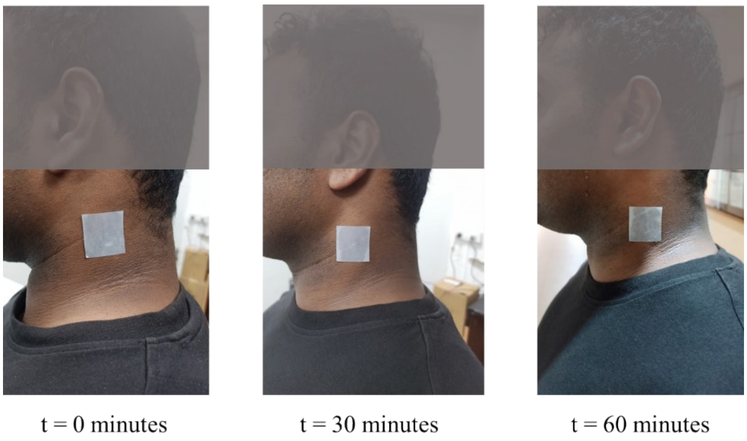

Figure S7 Photograph of adhesive patch showing good adhesion even after 30 and 60 minutes of intense exercise.

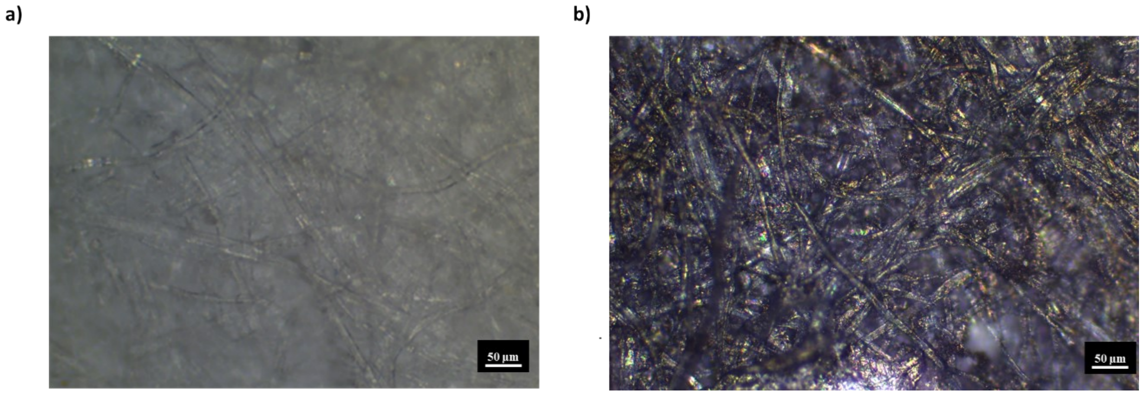

Figure S8 Optical microscopic image of the a) plane paper and b) nanoparticle-reduced paper

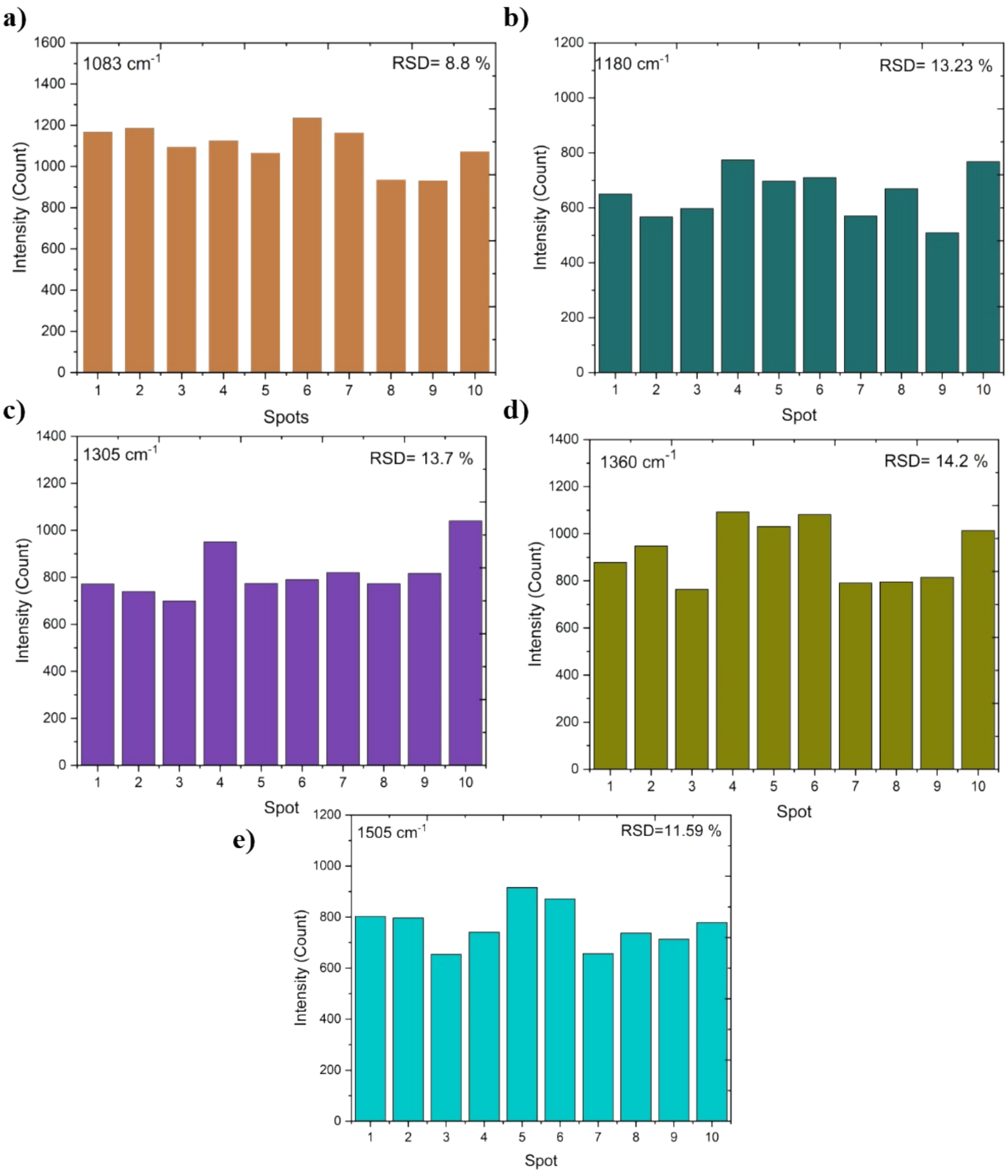

Figure S9 Evaluation of the Raman signal uniformity of Rh6G at different spots on the Au-reduced paper region for various peaks: a)1083 cm^{-1} b)1180 cm^{-1} c)1305 cm^{-1} d)1360 cm^{-1} and e)1505 cm^{-1}

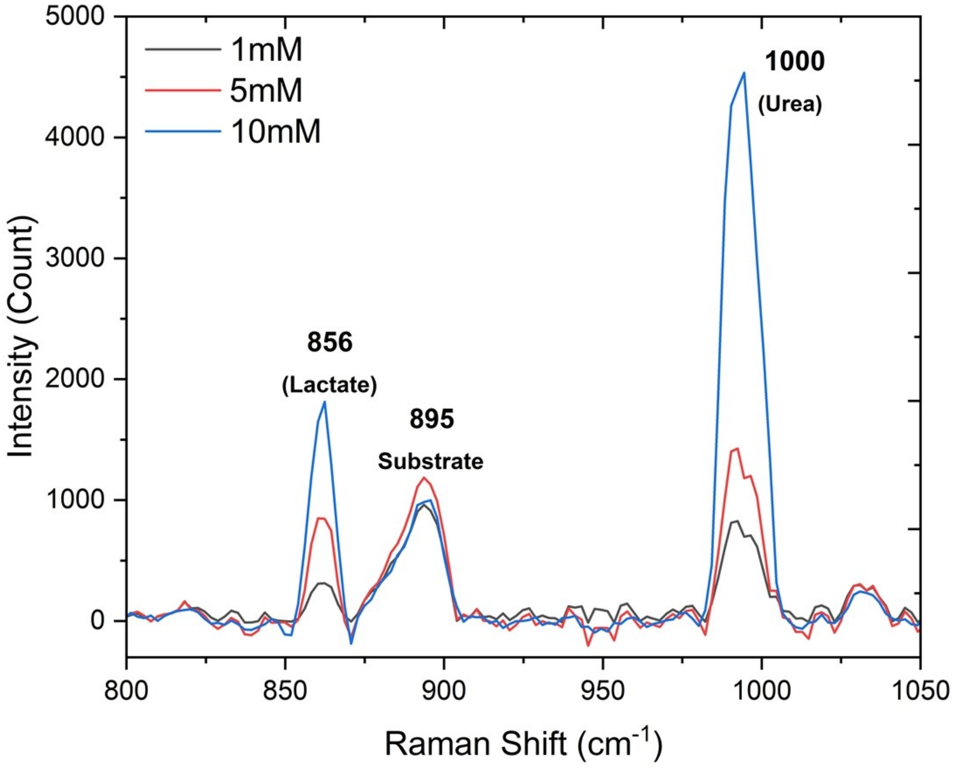

Figure S10 Raman spectra of mixture urea and lactate of different concentrations recorded on the developed substrate.

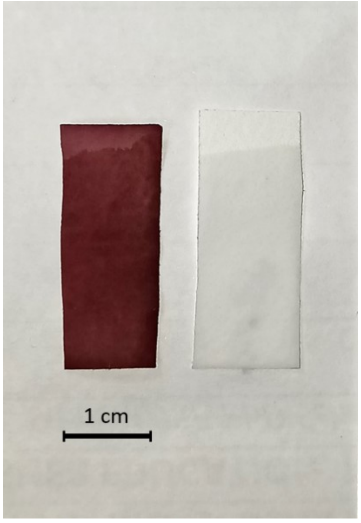

Figure S11 Comparison of the capillary height using normal filter paper and Au reduced plasmonic paper substrate.

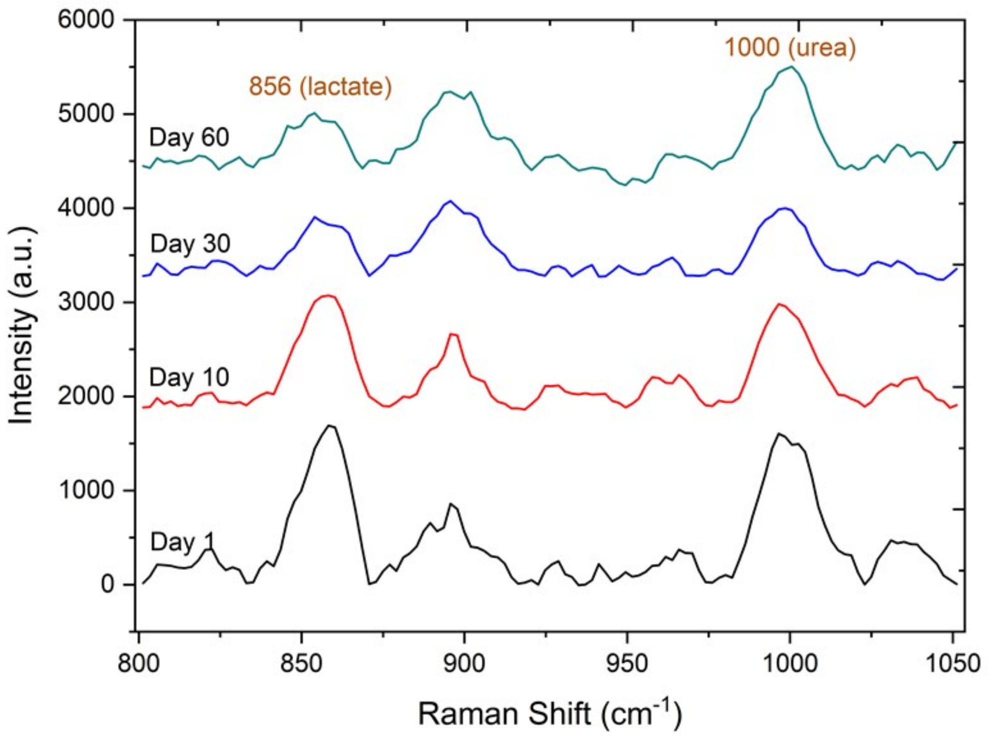

Figure S12 The Raman spectra of sweat recorded with the developed Au@PDMS/OA adhesive patch at different times.

Supplementary table ST1: Comparison of the analytical performance of the present work with similar reported SERS/Raman based sweat sensors.

No.	Substrate	Target analyte and LOD		Highlights	Drawbacks/Scope for improvements	Reference
		Urea	*Lactate*			
1	Wearable Janus fabric embedded with AgNPS	10µM	10µM	• Microstructured Optical Fiber (MOF) contributes to the enhancement of Raman signal and hence the good LOD<br>• Janus Textile Design ensures efficient sweat absorption and rapid transport to the sensing region<br>• High sensitivity and specificity, due to MOF and plasmonic nanostructures	• Optical fiber integration adds cost and complexity<br>• Less scalable<br>• May need additional modifications for multi-analyte detection	Han et al. [1]
2	Scotch tape based adhesive patch Au nanostars	0.6 µM	0.7 µM	• The plasmonic gold nanostars (GNS) helps in optimum SERS enhancement<br>• simultaneous detection of lactate, urea, and glucose<br>• Flexible & low-Cost adhesive tape based wearable patch for real-time monitoring	• Complexity in fabricating uniform sharp branched GNS and reproducibility<br>• The synthesis of anisotropic GNS requires careful control for batch consistency<br>• Durability of the adhesive tape-based substrate over the time	Supriya et al. [2]

3	PE film based flexible wearable patch with Au-Ag nanoshuttles (Au-Ag NSs)	2.3 µM	8.6 µM	• Au-Ag NSs for highly sensitive simultaneous detection of urea and lactate.<br>• Integrated a microfluidic system with capillary-driven flow for consistent and concentrated sweat collection<br>• The patch material is biocompatible and flexible	• Complexity in fabricating microfluidic based channels and hence the scalability<br>• Requires precise synthesis of Au-Ag NSs and reproducibility challenges<br>• Durability and wearability concerns for long time use	Yang et al. [3]
4	PDMS-PET based epidermal Optofluidic chip	0.4 mM	2.4 mM	• Wearable optofluidic patch for simultaneous detection of lactate, urea, and glucose in sweat.<br>• Eliminates the need for plasmonic nanostructures for enhancement<br>• Good repeatability even up to 90 days	• Lower Raman signal intensity compared to other SERS-based sensors<br>• Need of microfluidic fabrication, increasing complexity<br>• Concerns on adhesiveness on skin	Golparvar et al. [4]
5	Cotton fabric with Core-shell structured AuNR	-	50 µM	• wearable, thread-embroidered fabric microfluidic device for detection of lactate and glucose<br>• Core-shell structured gold nanorods (AuNRs@Au) as SERS tags results in high sensitivity and reproducibility.<br>• Successful validation on human volunteers at different body parts	• complex synthesis of core-shell AuNRs<br>• fabric material may loose adhesion onto skin upon long term use<br>• Relatively high complexity with need of embroidered microfluidic threads	Zhao et al. [5]
6	Au@PDMS/OA wearable adhesive patch (present work)	55 µM	47 µM	• Simple, biocompatible, wearable, adhesive and flexible patch, ensuring direct sweat absorption without the need for external microfluidic structures<br>• Simple and optimized fabrication approach which is easily scalable with good batch repeatability<br>• Nanoparticles in situ reduced onto cellulose fibers ensures reproducibility unlike colloidal NP based sensors	• Moderate and comparable LOD, but there is scope for improvement in terms of sensitivity<br>• Validation on more clinical samples could ensure more reliability<br>• Ideal for single use	Present work

1. Y. Han, X. Fang, H. Li, L. Zha, J. Guo and X. Zhang, *ACS Sensors*, 2023, **8**, 4774-4781.
2. S. Atta, Y. Zhao, S. Sanchez and T. Vo-Dinh, *ACS Omega*, 2024, **9**, 38897-38905.
3. H. Yang, Y. Ji, K. Shen, Y. Qian and C. Ye, *Biomedical Optics Express*, 2024, **15**, 14-27.
4. A. Golparvar, J. Kim, A. Boukhayma, D. Briand and S. Carrara, *Sensors and Actuators B*: *Chemica*l, 2023, **387**, 133814.
5. Z. Zhao, Q. Li, Y. Dong, J. Gong, Z. Li and J. Zhang, *Sensors and Actuators B: Chemical*, 2022, **353**, 131154.
